# Proton therapy in the most common pediatric non-central nervous system malignancies: an overview of clinical and dosimetric outcomes

**DOI:** 10.1186/s13052-019-0763-2

**Published:** 2019-12-27

**Authors:** Angela Sardaro, Roberta Carbonara, Maria Fonte Petruzzelli, Barbara Turi, Marco Moschetta, Arnaldo Scardapane, Amato Antonio Stabile Ianora

**Affiliations:** Interdisciplinary Department of Medicine, Section of Radiology and Radiation Oncology, University of Bari, p.zza Giulio Cesare nr.11, 70124 Bari, Italy

**Keywords:** Proton beam therapy, Radiation therapy, Pediatric non-central nervous system malignancies

## Abstract

Radiation therapy represents an important approach in the therapeutic management of children and adolescents with malignant tumors and its application with modern techniques – including Proton Beam Therapy (PBT) – is of great interest. In particular, potential radiation-induced injuries and secondary malignancies – also associated to the prolonged life expectancy of patients – are still questions of concern that increase the debate on the usefulness of PBT in pediatric treatments. This paper presents a literary review of current applications of PBT in non-Central Nervous System pediatric tumors (such as retinoblastoma, Hodgkin Lymphoma, Wilms tumor, bone and soft tissues sarcomas). We specifically reported clinical results achieved with PBT and dosimetric comparisons between PBT and the most common photon-therapy techniques. The analysis emphasizes that PBT minimizes radiation doses to healthy growing organs, suggesting for reduced risks of late side-effects and radiation-induced secondary malignancies. Extended follow up and confirms by prospective clinical trials should support the effectiveness and long-term tolerance of PBT in the considered setting.

## Introduction

Irradiation of primary or post-operative tumor site represents a fundamental part of the standard therapies for cancer due to the capability of X-rays to damage tumor cells DNA and induce tumor cells death [1]. Besides technological advances in photon beam radiotherapy (RT), potential long-term side-effects can affect neurocognitive and endocrine functions, as well as body-growth and fertility [2], in oncological pediatric patients. Indeed, it is well known that the use of RT in children and adolescents is particularly challenging [2] because of the increased risk of late toxicities (a serious concern in patients aged under 3 years [2, 3]) and secondary malignant neoplasms (SMNs) which are related to the higher radiation sensitivity and the increased cell-turnover of developing tissues [2].

Proton Beam Therapy (PBT) is a modality of charged particle therapy which provides excellent dose-distributions and an increased dose-sparing of normal tissues due to the absence of an exit-dose and an entrance-dose which is much lower than the target dose [1, 4]. These physical characteristics and the aforementionated dosimetric advantages suggest that PBT could be proposed as an alternative approach to conventional photon RT for the therapeutic management of malignant diseases [5, 6]. Furthermore, besides PBT benefits in normal tissues dose-sparing [3, 7–10], early clinical outcomes [4] also emphasized its advantages. Nevertheless, the question of PBT effectiveness and safety in comparison to modern high-conformal photon techniques is still debated [11, 12] due to the lack of long-term clinical data.

We observed that only selected papers analyzed particular critical issues related to proton treatments and their results were limited. Lodge at al [13]. considered that the use of protons for large tumors located next to critical organs at risk (OARs) was suggested, even if they did not investigated the role of PBT in pediatric patients [5]. Olsen [14] and Brada [15] summarized that PBT did not achieved sufficient evidences supporting its increased efficacy compared to other RT. Allen et al. [5] evidenced the benefit of PBT over photon treatments for pediatric Central Nervous System (CNS) tumors and other malignancies, but they underlined that existing data were still limited to provide conclusive recommendations for pediatric non-CNS tumors. In the *Consensus Report from the Stockholm Pediatric Proton Therapy Conference* published in 2016 [16], expert opinions on current indications for PBT in pediatrics confirmed that the majority of pediatric cancers which require RT should be treated with PBT. Protons were assumed as a preferred indication for most common pediatric CNS tumors, as well as for skull base tumors and retinoblastoma [16]. Nevertheless, there were different opinions on Hodgkin lymphoma – curable with lower radiation doses – and on rhabdomyosarcoma and Ewing sarcoma, for which the relative effectiveness of PBT depends on the tumor location [16].

Worldwide, the number of institutions offering PBT is gradually increasing and in the next years extensive data will provide more information on PBT effectiveness and cost-benefit rates in different scenarios.

We present a literature review of PBT performed for non-CNS pediatric tumors while specifically synthesizing its dosimetric and clinical advantages, which are changing the perspectives in radiation treatments. Whenever possible, we provided a discussion on emerging critical issues. We concluded this review with a summary of ongoing trials.

## Materials and methods

A PubMed search was carried out using the following Medical Subject Heading (MeSH) terms and arrangements: (((particle therapy[MeSH Terms]) OR proton beam therapy[MeSH Terms]) AND radiation therapy[MeSH Terms]) AND pediatric neoplasms[MeSH Terms]). Dosimetric comparison studies between PBT and photon-RT, as well as clinical studies and case series assessing outcomes of PBT in the most common pediatric non-CNS malignancies (retinoblastoma, Hodgkin lymphoma, sarcomas, Wilms tumor) were included in this review. We considered articles published in English from 2002 to 2018 with the aim to evaluate more recent data. Papers concerning other treatments, other neoplasms, socio-economic analyses, radiobiological and procedural issues, as well as review articles, editorials, consensus reports, modeling studies, case reports were excluded. Additional references from the retrieved review articles and consensus reports were also considered. Results were summarized and reported in relation to patients’ population and study assessment. Furthermore, for an overview of ongoing trials evaluating the application of PBT in the aforesaid setting, we reported an updating of studies currently registered on *clinicaltrials.gov* website.

## Results

**Fig. 1:**
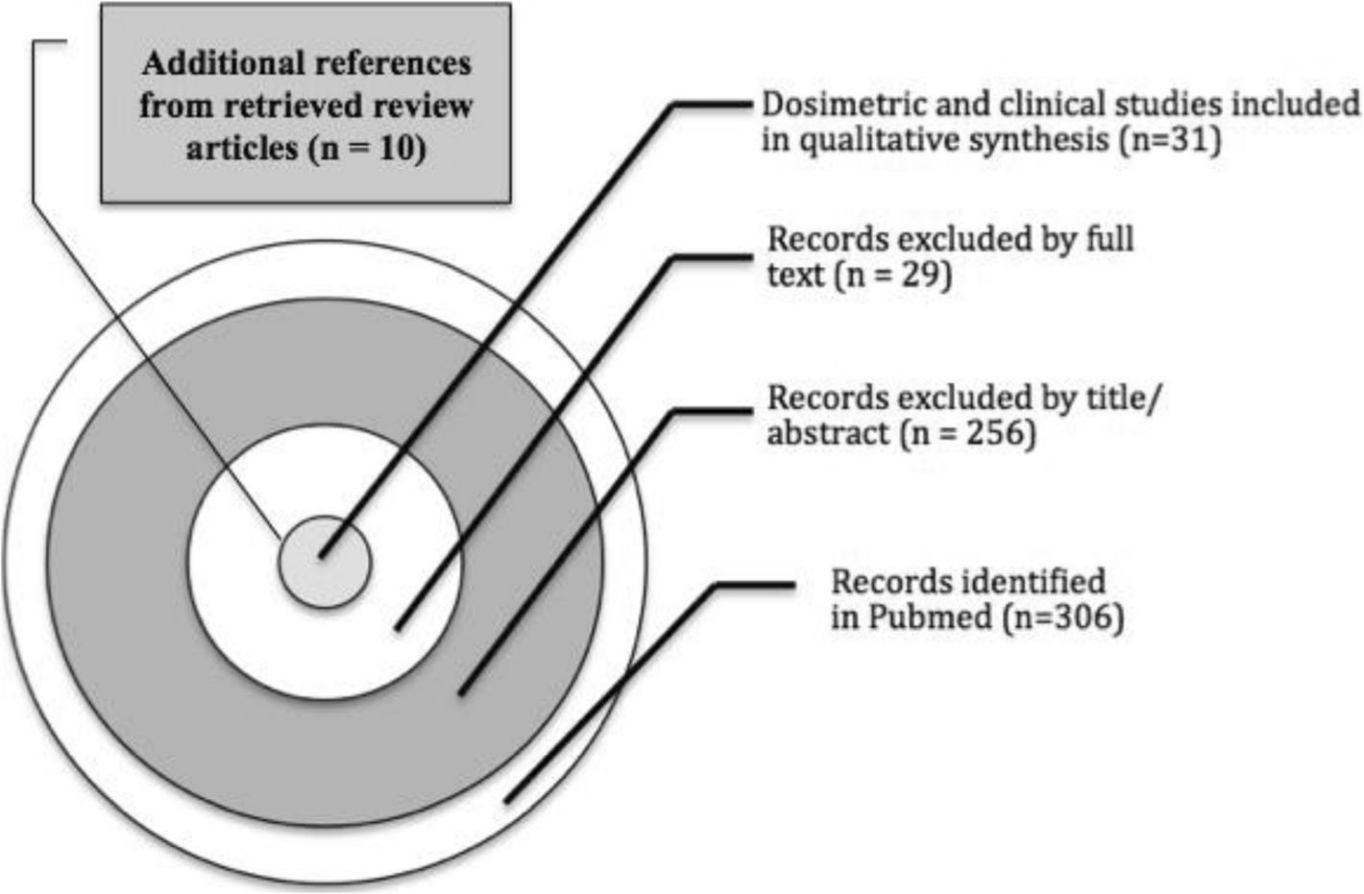
Study selection workflow

Among the reviewed papers satisfying the selection criteria, 31 articles – mainly reporting retrospective mono-institutional experiences – were included in a qualitative synthesis of dosimetric (Table 1) and clinical (Table 2) PBT outcomes (Fig. 1).

**Table 1:** Summary of literature describing dosimetric results achieved by PBT and comparison of PBT vs photons

Author (year)	Treatment planning study assessment	Number of PBT pediatric patients	PBT results
Retinoblastoma			
Krengli (2005) [17]	PBT with different beam arrangements/tumor locations; Isodose comparison, DVH analysis (for target and OARs)	–	Homogeneous target coverage, effective OARs-sparing. Potential reduction of SMNs and side effects.
Lee (2005) [18]	PBT vs 3D-CRT, electrons and IMRT; Isodose comparison, DVH analysis (target coverage and mean orbital volume receiving ≥5Gy)	3/8	Superior target coverage and orbital bone dose-sparing
Hodgkin lymphoma			
Andolino (2011) [19]	BS-PT vs 3D-CRT; DVH analysis (breast parameters); paired t-test	10	Significant reduction of dosimetric breast parameters
Hoppe (2012) [20]	INPT vs 3D-CRT and IMRT; Mean heart doses, mean doses to cardiac subunits; Wilcoxon paired t-test	2/13 total INPT patients (including adults)	Reduction of mean heart dose and mean doses to all major cardiac subunits (*p* < 0.05) (entire cohort)
Hoppe (2012) [21]	INPT vs 3D-CRT and IMRT; 50% reduction in the body V4; mean doses to OARs; paired t-tests	1/10 total INPT patients (including adults)	Reduced body V4 (*p* < 0.01) and mean doses to OARs (entire cohort)
Hoppe (2014) [4]^a^	INPT vs 3D-CRT and IMRT; integral body dose; mean doses to OARs	5/15 total INPT patients (including adults)	Reduced integral dose and mean doses to OARs (entire cohort)
Knäusl (2013) [22]	Treatment planning comparison (dosimetric parameters and DVHs for target and breast, thyroid, lungs, heart, bones) and SMNs assessment between PET-based RT with 3D-CRT, IMRT and PBT	10	The PET-based treatment planning ensures dosimetric advantages for OARs. PBT can further improve these results in terms of toxicity risk reduction
Soft tissue sarcoma			
Weber (2004) [23]	IMPT vs IMRT, dose-escalated IMPT; DVH analysis (for target and OARs), inhomogeneity coefficient, conformity index	5	Similar level of tumor conformation, improved homogeneity with mini-beam IMPT, substantial reduction of OARs integral doses, dose-escalation always possible
Rhabdomyosarcoma			
Miralbell (2002) [24]	PBT, IMPT vs conventional RT and IMRT; model-based SMNs risk assessment	1/2	Reduction of SMNs risk by a factor of ≥2
Ladra (2014) [25]	PBT vs IMRT; dosimetric parameters for target and OARs; paired t-tests, Fisher’s exact test	54	Comparable target coverage (*p* = 0.82). Reduced mean integral dose. Significant sparing for 26 of 30 OARs (p < 0.05)
Kozak (2009) [26]	PBT vs IMRT; dosimetric parameters for target (target covarage and dose-conformity) and OARs two-tailed, Wilcoxon signed-rank test	10	Acceptable and comparable target coverage. Significant superior OARs-sparing, except for ipsilateral cochlea and mastoid / borderline significance for ipsilateral parotid (*p* = 0.05)
Cotter (2011) [27]	PBT vs IMRT; dosimetric parameters for target and OARs Wilcoxon signed-rank test	7	Comparable target coverage. Significant reduction in mean OARs dose (*p* < 0.05) and bone volume receiving > 35 Gy
Lee (2005) [18]	PBT vs 3D-CRT and IMRT; Isodose and dose-volume comparison for target and OARs	3/8	Superior target coverage and OARs dose-sparing (0% of mean ovarian volume received ≥2 Gy)
Yock (2005) [28]	PBT vs 3D-CRT; DVH analysis for OARs (orbital and CNS structures)	7	Superior OARs dose-sparing
Wilms tumor			
Hillbrand (2008) [29]	Passively scattered/scanned beams PBT vs conventional RT and IMRT; DVH analysis (liver and kidney dosimetric parameters); model-based SMNs risk assessment	4/9	Superior dose-sparing for liver and kidney (mean liver and kidney dose reduced by 40–60%). Reduced SMNs risk with scanned beams PBT

*DVH* Dose-volume histogram, *SMNs* Second malignant neoplasms, *BS-PT* Breast-sparing proton therapy, *INPT* Involved-node proton therapy

^a^Studies by Hoppe based on the patients cohort enrolled in an institutional review board-approved protocol at the University of Florida Proton Therapy Institute

**Table 2:** Summary of literature describing clinical outcomes of PBT in the most common pediatric non-CNS malignancies

Author (year)	Method	Number of PBT pediatric patients	Med FU # mo or y (range)	Med Total Dose # CGE or Gy (RBE) (range)	Combined treatments	Outcomes #
Retinoblastoma						
Sethi (2014) [12]	R/C (protons vs photons)	55/86	6.9 y (1–24.4)	44.16 Gy (RBE) (40.0–50.0)	Variable ** (chemotherapy)	10y cumulative incidence of in-field SMNs: 0% (vs 14% with photons, *p* = 0.015)
Mouw (2014)^a^ [30]	R	49 (60 eyes)	8 y (1–24)	44.0 Gy (RBE) (40–46.8)	Variable ** (chemotherapy, cryotherapy/laser)	Enucleation-free survival: 81.6% No in-field SMNs
Hodgkin lymphoma						
Hoppe (2014) [4]	P	5/15 (mix A-P patients)	37 mo (26–55)	15–25.5 CGE	Variable ** (chemotherapy)	3y RFS: 93% (1 relapse among pediatrics) 3y EFS 87% No acute or late grade ≥ 3 toxicities
Wray (2016) [31]	R	22	36 mo	21 Gy (RBE; range, 15–36) including 9 patients treated with a sequential boost due to an incomplete response	Variable ** (chemotherapy)	2-year and 3-year OS rates: 94%, 2-year and 3-year PFS rates were both 86%. 3 high-risk patients recurred. No acute or late grade ≥ 3 toxicities
Chordoma/Chondrosarcoma						
Hug (2002) [32]	R	13/29 (mix benign-malignant)	40 mo (13–92)	CH: 73.7 CGE (70–78.6) CS: 70.0 CGE (69.6–70.2)	Variable ** (surgery; protons-photons)	5y LC*: 60% CH, 100% CS 5y OS*: 60% CH, 100% CS 2% severe late effects
Habrand (2008) [33]	R	30	26.5 mo (mean)	68.4 CGE (54.6–71) (Mean total dose for CS/CH)	Variable ** (surgery; protons-photons)	5y OS: 81% CH, 100% CS 5y PFS: 77% CH and 100% CS Grade 2 late toxicity: 7 patients; grade 3: 1 patient
Rutz (2007) [34]	R	3/26 (mix A-P patients)	35 mo (13–73)	CH: 72 CGE (59.4–74.4)	Variable ** (surgery; photon RT)	3y OS*: 84% 3y PFS*: 77% Late toxicity: 4 patients
Rutz (2008) [35]	R	10	36 mo (8–77)	CH: 74 CGE CS: 66 CGE (63.2–68)	Variable ** (surgery; chemotherapy)	LC, OS and FFS: 100% Late toxicity: grade 1 (2 patients), grade 2 (1 patient)
Ares (2009) [36]	R	64 (mix A-P patients)	38 mo (mean) (14–92)	CH: 73.5 RBE CS: 68.4 RBE	Variable **	5y LC*: 81% CH and 94% CS 5y DSS*: 81% CH and 100% CS 5y OS*: 62% CH and 91% CS high-grade toxicity: 4 patients
Staab (2011) [37]	R	3/40 (mix A-P patients)	43 mo (24–91)	CH: 72.5 Gy (RBE) (mean total dose) (59.4–75.2)	Variable ** (surgery; protons-photons)	5y LC*: 62% 5y DFS*: 57% 5y OS*: 80% (rates were 100% without SS)
Rombi (2013) [38]	R	26	46 mo (mean) (4.5–126.5)	CH: 74 RBE (73.8–75.6) CS: 66 RBE (54.72)	Variable ** (surgery)	5y LC*: 81% CH and 80% CS 5y OS*: 89% CH and 75% CS No high-grade late toxicities
Soft tissue sarcoma						
Timmerman (2007) [39]	R	16 (various histologies)	18.6 mo (4.3–70.8)	50 CGE (46–61.2)	Variable ** (surgery, chemotherapy)	LC: 75% 1y PFS: 81.8% 2y PFS:71.6% 1y OS: 90.9% 2y OS: 69.3% Mild acute toxicity (G3-G4 in bone marrow with concurrent chemotherapy)
Rhabdomyosarcoma						
Ladra (2014) [25]	P	54	3.9 y	Variable according to tumor site 45–50.4 Gy (RBE)	Variable **	3y EFS: 69%; 5y EFS: 65% 3y OS: 80%; 5y OS 77% 3y LC: 78%; 5y LC: 78% Late grade 3 toxicity: 3 patients / No SMNs
Leiser (2016) [40]	R	83	55.4 mo (0.9–126.3)	54 Gy (RBE) (41.4–64.8)	Variable ** (chemotherapy)	5y LC: 78.5% (95% CI, 69.5–88.5%) 5y OS: 80% (95% CI, 71.8–90.0%) 5y grade 3 toxicity: 3.6% No grade 4–5 toxicity SMNs: 1.2% (1/83) Quality of life significantly increased
Childs (2012) [41]	R	17	5 y (2–10.8)	50.4 Gy (RBE) (50.4–56.0)	Variable ** (chemotherapy, photon RT, surgery)	5y-FFS: 59% (95% CI, 33–79%), 5y-OS: 64% (95% CI, 37–82%). 5y-Late effects in 10 patients (58.8%)
Cotter (2011) [27]	R	7	27 mo (10–90)	36–50.4 CGE	Variable ** (surgery, chemotherapy)	71% of patients with no evidence of disease Good treatment tolerance No SMNs
Yock (2005) [28]	R	7	6.3 y (3.5–9.7)	46.6 CGE (40–55)	Variable ** (photon RT, chemotherapy)	DFS: 100%, LC: 6/7 patients (85%) Excellent orbital functional outcome
Weber (2016) [42]	R	39	Mean 41 mo (9–106 mo)	54 Gy (RBE) (50.4–55.8)	Neoadjuvant and concomitant chemotherapy	10 patients failed. PFS*: 72% (95% CI, 67–94%), 5-year OS: 73% (95% CI, 69–96%). A delay in the initiation of PT (> 13 weeks) was a significant detrimental factor for PFS. 3 (8%) patients had grade 3 toxicity (eye/ear). 5-year grade 3 toxicity free survival*: 95% (95% CI, 94–96%)
Vern-Gross (2016) [43]	R	66	1.5 y	50.4 Gy (RBE)	Chemotherapy	2-year LC* and OS*: 88 and 89%. Permanent toxicity affected only 9 pts. (eye, ear, ormonal). Median survival after initial recurrence was 6 months (range:1–25)
Mizumoto (2018) [44]	R	55	24.5 mo (1.5–320)	50.4 GyE (36.0–60.0)	Variable ** (surgery, chemotherapy)	1- and 2-year OS rates were 91.9 and 84.8% 1- and 2-year PFS rates were 81.6 and 72.4% 1- and 2-year LCRs were 95.6 and 93.0% 13 patients recurred Grade > 3 late toxicities were not occurred
Ewing sarcoma						
Rombi (2012) [45]	R	30	38.4 mo (17.4 mo - 7.4 years)	54 Gy (RBE) (45–59.4)	Variable ** (surgery; chemotherapy)	3y EFS*, 60% 3y LC*: 86% 3y OS*: 89% Mild/moderate acute skin toxicity 4 hematological SMNs with combined chemotherapy

# If not specifically reported, results are referred to entire cohort when mixed population is considered

RBE: relative biological effectiveness, CGE: cobalt Gray equivalent;

*FU* Follow-up, *A-P* Adult-pediatric

*R* Retrospective, *C* Comparative, *P* Prospective

**variable: different surgery/chemotherapy/RT approaches performed in the patient population

*LC* Local control, *OS* Overall survival, *PFS* Progression free survival, *RFS* Relapse-free survival

*EFS* Event-free survival, *FFS* Failure-free survival, *DSS* Disease specific survival, *DFS* Disease free survival

*SS* Surgical stabilization

* actuarial rate

*CI* Confidence interval

^a^Studies by Sethi et al. and Mouw et al. based on the same patients cohort treated with PBT at Massachusetts General Hospital between 1986 and 2012 [46]

### Retinoblastoma

Retinoblastoma (RB) represents the most common primary ocular malignancy in childhood and it typically affects children under 4 years [17]. Patients often present a germ-line mutation of RB1 tumor suppressor gene. RT was used in selected patients to avoid surgical enucleation, even if long-term RT side effects such as conjunctivitis, corneal opacification, cataract, glaucoma, vitreous hemorrhage, retinopathy, optic neuropathy, orbital hypoplasia were observed [17, 47, 48]. Furthermore, although RB is a radiosensitive tumor, the use of RT could increase the risks of radiation-induced SMNs [12, 49], which is greater in children with hereditary RB gene mutation [12, 17, 50]. For these reasons, RT is currently considered as a salvage-therapy and modern therapeutic eye-preserving approaches include crio-ablation, laser, chemotherapy [11, 17, 49]. Various high-conformal RT techniques (fractionated stereotactic RT, intensity-modulated radiotherapy (IMRT), PBT) have been adopted to spare OARs [17, 49] and to reduce radiation side-effects [17]. PBT represents the most conformal external-beam RT option currently available for RB [30], since it reduces the integral dose to healthy tissues by depositing the majority of energy in the “Bragg peak” [17].

Krengli et al. [17], in their study on the optimization of proton-beam arrangements for various intra-ocular tumor locations, concluded that PBT could reduce the risk of radiation-induced SMNs and cosmetic and functional side-effects due to its dosimetric benefits (Table 1). In the treatment planning study presented by Lee et al. [18], (Table 1), PBT achieved the best target-coverage (which clinically might translate into a reduced risk of tumor recurrence) and orbital bone dose-sparing compared to photon-RT techniques.

Long-term tumor control and toxicity outcomes after PBT were investigated by Mouw et al. [30] (Table 2) among patients treated for early or locally-advanced disease. A high disease-local control (LC) was observed during a prolonged follow up period both in early and advanced cases, with no patients died for RB or developing metastatic disease; treatment-related ocular side-effects were uncommon, many patients retained useful vision in the treated eye and no SMNs were observed [30]. As focused in the PTOG/PROS/EPTN (*Particle Therapy Co-Operative Group/Pediatric Radiation Oncology Society/European Particle Therapy Network*) consensus statement [11], data by Mouw et Al. suggest that PBT should be reconsidered for early-stage patients, even if global evidences on the reduction of SMNs-risk using PBT are still low [46]. For this reason, although PBT dosimetric advantages suggest its safety [12, 48, 50], confirms from further studies with a long-term follow up are necessary.

### Hodgkin lymphoma

Hodgkin lymphoma (HL) is a malignancy which usually affects adolescents and young adults [20] with a 5 years-overall survival (OS) rate varying in the range between 85 and 95%, in relation to disease stage and prognostic factors present at diagnosis [19, 51, 52]. Combined-modality treatment regimens for early-stage HL integrate RT for its benefits in loco-regional disease control and overall outcome [53]. In advanced-stages, adjuvant RT is used for patients treated with less-intensive chemotherapy regimens or with incomplete or slow responses to chemotherapy [54, 55]. Despite RT advantages in tumor control, an increased risk of treatment-related chronic toxicity in survivors have been observed. In particular, RT on mediastinum combined with anthracyclines increases the risk of cardiovascular disease (such as coronaropathy, valvular diseases, cardiomyopathy, arhythmia, pericarditis) and cardiac death as late sequelae [20, 56]. Also an increased incidence of secondary breast cancer in female survivors has been reported [19, 57, 58]. In summary, an elevated risk of developing SMNs and cardiovascular diseases have been observed as late side-effects even 30 years after chemo-radiation [21, 56] and at lower RT threshold doses [20].

When both chemotherapy and RT are necessary to treat HL, the risk of toxicity for normal tissues could be limited minimizing treatment field size and reducing radiation doses in combined-modality regimens [20, 55, 59, 60]. High-conformal RT techniques – such as IMRT combined with sophisticated systems for image-guidance or PBT – also allow to reduce the risk of RT-related late effects, with substantial benefits especially in young HL patients which have high survival rate [20]. Indeed, IMRT improves OARs dose-sparing in the high-dose region because of its capability to shape the dose distribution around concave structures. However, an important IMRT disadvantage consists in the exposure of OARs to low radiation doses [19].

Andolino et al. [19] (Table 1) compared breast-sparing proton therapy (BS-PT) with involved-field 3D-CRT for pediatric female HL patients and concluded that this technique was able to markedly reduce (by 80%) breast dose. Hoppe et al. [4, 20, 21] enrolled patients (including adults, children and adolescents) with Stage IA-IIIB HL and mediastinal involvement on a prospective study comparing adjuvant involved-node proton therapy (INPT) with 3D-CRT and IMRT. They observed [4, 21] that PBT provided the lowest mean dose to heart, lungs and breasts for all patients (Table 1), with an improvement in dose-sparing also for esophagus and thyroid. Authors also reported [20] the reduction of radiation doses to all major cardiac subunits with PBT, suggesting for a potential limitation of late cardiac toxicities, even if confirms in long-term follow-up were necessary [20]. Another issue assessed by Hoppe et al. [4] concerns the early clinical outcomes (Table 2) of performed INPT: treatments were well-tolerated, with disease outcomes similar to those obtained with conventional photon-RT; nevertheless, a long-term follow-up was considered necessary to evaluate the likely benefit of PBT in reducing the risk of late toxicity [4]. Similar findings were reported by Wray [31] and other authors of the same research group at the University of Florida Health Proton Therapy Institute after the clinical outcomes assessment of 22 HL patients treated with PBT.

To our knowledge, to date, no randomized studies have assessed long-term effectiveness and tolerance of INPT versus INRT/involved-site (IS) RT – newer photon-RT approaches with reduced fields/volumes which are becoming the new standard of care in radiation treatments for HL in the era of modern imaging [53, 61].

To conclude, as reported also by Weber et Al. [11] – who revised the *Evidence-based Review on the Use of Proton Therapy in Lymphoma From the Particle Therapy Cooperative Group (PTCOG) Lymphoma Subcommittee* [62] – the dosimetric advantages of PBT are expected to translate into a lower risk of late toxicities and SMNs, establishing the foundation for PBT clinical application and further researches to confirm the expected outcomes.

### Sarcoma

#### Chordoma and Chondrosarcoma

Chordoma (CH) and chondrosarcoma (CS) are uncommon neoplasms in children with relatively low metastatic potential [37, 63]. CH can occur along the axial skeleton, usually at the skull base or near the coccyx [11, 37]. CS can involve the pelvis or long bones, with rare presentation at the skull base [11]. Surgery is considered the first-line therapeutic approach [11], however total resection can rarely be achieved because of tumor proximity to critical structures [32, 36]. Also RT planning is limited by OARs tolerance: thus, the delivered RT doses could result in a suboptimal long-term tumor control [32, 64]. Anyhow, because of the low metastatic potential of these tumors, LC, OS and progression free survival (PFS) are very important aspects and adjuvant radiation treatments remain recommended. In this setting PBT has established itself as an optimal approach, especially for skull-base CH and CS [36].

Hug et al. [32] (Table 2) analyzed children with mesenchymal tumors invading the skull base (including CH and CS) which were treated with fractionated proton or combined proton-photon therapy: their data suggested that PBT delivered after a major skull base surgery could offer advantages in tumor control and survival. Also Habrand et al. [33] observed excellent LC and low-grade late toxicities after high-dose photon-proton therapy performed in a similar setting (Table 2).

Rutz et al. [34] evaluated patients with extra-cranial CH (Table 2) treated with postoperative spot-scanning PBT^1^ (performed after a function-preserving surgery) and observed high OS and PFS rates with acceptable treatment tolerance. In a consecutive study, Rutz et al. [35] (Table 2) reported satisfactory preliminary outcomes of postoperative spot-scanning PBT delivered in combination with or without intensity-modulated proton therapy (IMPT). PBT was well tolerated with late adverse events (mild to moderate in degree) reported only in three cases.

The effectiveness of spot-scanning PBT for extra-cranial CH was also evaluated by Staab et al. [37] for adult and pediatric patients (Table 2). In this study, patients with gross residual disease before PBT and no surgical stabilization (SS) obtained a 100% LC rate at 5 years; among patients who underwent prior titanium-based SS and reconstruction of the axial skeleton, a significant reduction (30%) of 5-year LC rate was observed. Authors concluded that PBT achieved high rates of tumor LC – even for large, unresectable diseases – which were significantly better in patients without SS. Also Rombi [38] and Ares [36] confirmed excellent outcomes and acceptable late toxicities using fractionated spot-scanning PBT (Table 2).

As previously observed in the PTOG/PROS/EPTN consensus [11], since CH and CS are radioresistent tumors which require high target doses, PBT could represent an ideal approach to provide target dose-escalation with reduced overall integral dose. The results detailed in our literature review (Table 2) are in line with data summarized by Weber et Al. [11], who reported the 5-years OS after PBT in the range 68–89%.

#### Soft tissue sarcoma

RT plays an important role in the multimodal management of childhood sarcoma [50]. For resectable sarcoma the standard-of-care is surgery followed by adjuvant RT for higher-risk patients, while for unresectable tumors neoadjuvant chemo-RT followed by surgery and adjuvant chemotherapy is indicated [66]. A preliminary multidisciplinary evaluation is recommended [66]. Nevertheless, the proximity to dose-limiting OARs (as occurs in head and neck, parameningeal or paraspinal tumor localization) could influence the radiation treatment choice. Especially in these cases, PBT could represent a valid alternative RT approach to preserve patients quality of life by reducing OARs doses.

In Weber’s treatment planning study [23] – which compared IMRT and IMPT for paraspinal sarcoma – an increased dose-sparing for OARs and a potential target dose-escalation were reported with PBT.

Timmerman et Al. [39] (Table 2) investigated the feasibility of spot-scanning PBT in children with soft tissue sarcoma (including rhabdomyosarcoma) arise in critical sites and unresectable in the majority of cases: PBT was feasible and well tolerated, with early LC comparable to outcomes reported with conventional RT.

#### Rhabdomyosarcoma

Rhabdomyosarcoma (RMS) is the most common soft-tissue sarcoma in childhood which represents a highly-malignant and locally-invasive neoplasm [41]. This tumor commonly occurs in sites within head and neck presenting also parameningeal locations [67]. A potential reduction of radiation-induced SMNs using PBT was suggested by a model-based secondary cancer risk assessment performed by Miralbell et al. [24]. PBT has also provided dosimetric and clinical advantages, as highlighted in the following summarized studies (Tables 1 and 2).

Ladra et Al. [25] published the first comparison of proton vs photon dosimetry from a prospective multi-institutional phase II clinical trial which enrolled 54 children (the first largest published dosimetric series for RMS); they demonstrated a lower integral dose, an increased OARs dose-sparing and satisfactory clinical outcomes with passively-scattered PBT. Leiser et Al. [40] observed minimal late toxicity and improved quality of life in 83 RMS children treated with pencil beam scanning PBT combined with chemotherapy. Confirms of the short-term effect and acute tolerability of PBT derive from a recent published experience among 55 RMS patients in Japan [44].

RT and chemotherapy represent the main treatment choices for parameningeal rhabdomyosarcoma (PRMS). Kozak et al. [26] compared PBT and IMRT plans for pediatric PRMS: both plans allowed acceptable and comparable target-coverage, but the higher dose-conformity provided by PBT resulted in a significant improvement of dose-sparing for the majority of the considered OARs. Childs [41] reported tumor LC and survival rates after PBT comparable to those in literature controls. Weber et al. [42] (Table 2) reported the clinical outcomes of 39 pediatric PRMS patients treated with PBT and chemotherapy, evaluated over a median follow-up of 41 months: authors observed encouraging results, in line with the previously reported evidences on the safety and effectiveness of PBT in the considered setting [42].

Cure rates for genitourinary (e.g. bladder/prostate) RMS with a multimodal approach are 70 to 80%, but significant late side-effects are often observed [27]. Cotter et al. [27] reported comparable target dosimetry between PBT and IMRT in this setting, but PBT led to a significant decrease in mean dose to the considered OARs (bladder, testes, femoral heads, growth plates and pelvic bones), suggesting for a reduced risk of late toxicity. Lee et al. [18] compared dose-distributions and dose-volume histograms (DVHs) of 3D-CRT, electron-RT, IMRT and PBT plans for three pediatric disease sites, including pelvic sarcomas: PBT was superior in eliminating any dose to the ovaries and reducing doses to the pelvic bones and vertebrae. Due to these advantages, PBT in pelvic sarcoma could have the potential clinical benefit of preserving reproductive and hormonal functions, as well as body growth. Confirms from extended clinical data are necessary.

Pediatric patients with orbital RMS often receive combined chemotherapy and RT [28]. However, late effects of photon RT can potentially affect functional and cosmetic results [28]. PBT has shown to provide excellent target dose-distributions with increased OARs dose-sparing. Indeed, Yock et al. [28] reported PBT advantages in target-coverage and doses to brain, pituitary gland, hypothalamus, temporal lobes and ipsilateral/contralateral orbital structures as compared to 3D-CRT, even if tumor size and location affected the degree of OARs dose-sparing. Local and distant tumor control with PBT compared favorably to previous published results [28].

Available dosimetric and clinical results of PBT for RMS are promising. As considered also by Weber et Al. [11], PBT could offer an alternative therapeutic approach that should be able to reduce side effects on developing organs.

#### Ewing’s sarcoma

Ewing’s sarcoma is a rare malignant bone and/or soft tissue small blue round cell cancer [11], often occurring in sites that are not easily resected [45]. It is highly responsive to RT, which has been widely used [45]. In particular, as summarized by Rombi [45, 50], RT is prescribed in the postoperative setting (for patients with close or positive resection margins and in cases with a poor or slow clinical response to neoadjuvant chemotherapy) or in children with unresectable tumors/higher risk of post-surgical morbidity. Nevertheless, also definitive RT produces side-effects as a result of normal tissues exposure [45] and PBT has been suggested as an alternative treatment option to reduce this risk [45, 68].

In particular, Rombi et al. [45] reported (Table 2) good clinical outcomes and treatment tolerance among 30 children treated with PBT, supporting the premise that protons could be used similarly to photons to achieve analogous disease control rates at comparable doses. Even if available data of PBT application in this setting are still limited to produce significant evidences [46], the main clear advantage of protons is to reduce normal tissue doses. The potential reduction of late toxicities and the possibility of target dose-escalation [11, 68] could represent consequent benefits.

## Wilms tumors

In patients affected by Wilms tumor – the most common childhood renal neoplasm [11] – RT is indicated to improve LC when incomplete resection, higher stage or unfavorable histology occur [11].

Few studies on PBT in this setting are reported. Hillbrand et al. [29] described a substantial reduction in mean liver and kidney dose with PBT compared to conventional RT and IMRT (Table 1). SMNs risk with scanned beams-PBT was inferior to IMRT and passively-scattered PBT (which is associated to secondary neutrons production) (Table 2). Also the PTOG/PROS/EPTN consensus statement [11] has underlined the improvement in OARs dose-sparing with PBT, even if further evaluations on long-term local failure and patients survival are required.

## Ongoing trials

Among the ongoing trials on PBT in pediatric patients registered (*clinicaltrials.gov*) at the time of this review, fifteen were analyzing outcomes of PBT in the discussed non-CNS malignancies: six were active but not recruiting, five were in recruiting status, one was completed and three were terminated.

**Fig. 2:**
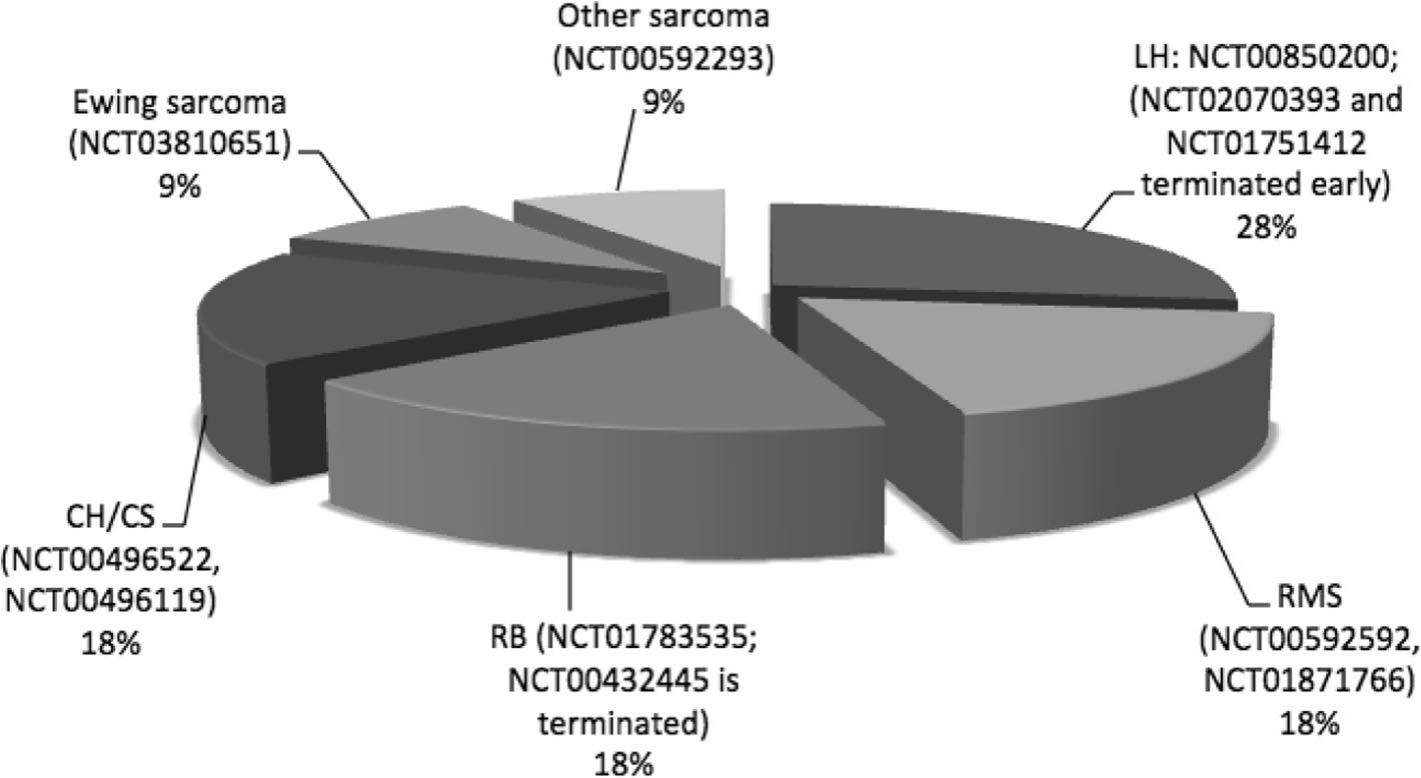
Ongoing trials evaluating specific primary pediatric non-CNS tumors

Figure 2 regroups 11 trials which were evaluating specific pediatric non-CNS tumors.

Six studies were specifically assessing dosimetric issues (NCT01502150, NCT02070393, NCT00592592, NCT00850200, NCT00592293, NCT01751412). The main goal of these prospective study is to obtain information on PBT effectiveness and toxicity, as well as wider dosimetric data from treatment plans, with the aim to improve the planning and delivery of PBT for future approaches in pediatric patients.

## Discussion

We present a summary of dosimetric and clinical results achieved with PBT in pediatric patients affected by non-CNS tumors.

At the time of our review, recent authoritative reports [11] have suggested that existing clinical data is still too preliminary to add new directions in clinical practice. Nevertheless, dosimetric advantages of PBT over photon techniques were well-known and knowledge of charged particles’ physical characteristics was consolidated. Basing on the concept that dosimetric data could lead to a prediction of clinical outcomes, we tried to assess if – in particular cases – PBT dosimetric benefits could translate into clinical gains.

In primis, our research confirmed that PBT improves OARs dose-sparing; additionally, PBT with spot-scanning/pencil beam scanning and IMPT modalities reduce neutron dose-contamination. These are crucial topics that increase radiation oncologists’ interest on PBT application in pediatric treatments, especially when radioresistent tumors arise next to critical anatomic sites require higher radiation doses or a dose-escalation [23, 26] (e.g. paraspinal and parameningeal sarcoma).

The overall actual clinical results are confirmed as not-exhaustive to provide high-level evidences in all indications [11, 46]. Among the retrieved articles, only few studies [12, 30] reported long-term clinical data. Despite early tumor control and patients survival rates with PBT resulted high (Table 2), long-term clinical results are mandatory to assess treatment effectiveness and tolerance in pediatric cohorts.

Costs and difficulties to access to PBT centers (depending on the small number of centers able to perform PBT) are still elevated [69] and could affect the prospect to provide significant evidences on large sample sizes. To overcome these difficulties, comprehensive database or wider registries [11] could support analyses aimed to define the indications for PBT in the multidisciplinary management of pediatric non-CNS tumors.

Careful analyses have also to be performed to balance PBT cost-effectiveness with requests of managing potential late effects related to different RT approaches.

Moreover, further studies with appropriate methodologies are required to answer to actual recurring questions, such as: What differences in OARs-sparing and tolerance profiles can be observed between PBT and modern high-conformal photon techniques supported by sophisticated image-guidance systems [5]? Which potential outcomes/effects could be associated to PBT combined with new drugs [11]? Is radiobiology of protons clearly understood [6, 11, 68]? Indeed, one major limitation of previous published works – besides the retrospective nature of their observations – was the comparison between PBT and photon-RT with no advanced technologies [11, 12]. Modern technologies for image-guidance^2^ and treatment planning (e.g. image-guided IMRT on limited volumes) have improved dose-conformity and OARs dose-sparing even with photon-therapy [11, 49]. Nevertheless, clinical results of modern photon-IGRT are still limited. Additionally, in IMRT treatments, developing OARs remain at risk of non-target radiation dose [11] and could be at increased risk of SMNs as compared to conventional 3D-CRT [2] due to low radiation doses [1].

In 2018 one of the first treatment planning comparison between IMPT and highly sophisticated deep-inspiration breath hold Volumetric Modulated Arc Therapy (VMAT) in adults has been published [71]: even if VMAT was planned in a very experienced center, IMPT provided higher target coverage and reduced mean doses to the considered OARs [71]. These results are promising and could produce implications in pediatric research, inducing radiation oncologists to further consider PBT application and promote clinical trials.

## Conclusion

Since the long life expectancy of patients is a major issue in oncological pediatric treatments, adequate analyses on RT late effects become necessary. PBT has provided dosimetric advantages for normal tissues as compared to photon-RT, but long-term clinical results and comparisons with modern photon-RT outcomes are still required. Ongoing and future investigations should clearly define the role of PBT in the multimodal management of the most common pediatric non-CNS tumors.

## Data Availability

All data generated or analysed for this review are included in this published article.

## Abbreviations

3D-CRT: Three-Dimensional Conformal Radiotherapy
BS-PT: Breast-sparing proton therapy
CH: Chordoma
CNS: Central Nervous System
CS: Chondrosarcoma
DVHs: Dose-volume histograms
HL: Hodgkin lymphoma
IMPT: Intensity-modulated proton therapy
IMRT: Intensity-modulated radiotherapy
INPT: Involved-node proton therapy
ISRT: Involved-site radiotherapy
LC: Local control
OARs: Organs at risk
OS: Overall survival
PBT: Proton Beam Therapy
PFS: Progression free survival
PRMS: Parameningeal rhabdomyosarcoma
PTOG/PROS/EPTN: *Particle Therapy Co-Operative Group/Pediatric Radiation Oncology Society/European Particle Therapy Network*
RB: Retinoblastoma
RMS: Rhabdomyosarcoma
RT: Radiation therapy (or radiotherapy)
SMNs: Secondary malignant neoplasms
SS: Surgical stabilization
VMAT: Volumetric Modulated Arc Therapy

## Footnotes

1 Spot-scanning is a PBT delivery method which provide a uniform and complex dose-distribution with the advantage of reduced neutron contamination [65]

2 Nowadays image-guidance is being used in association with current knowledge on optimization/reduction of exposure to imaging doses [70]
